# Effective assay technologies fit for large-scale population screening of type 1 diabetes

**DOI:** 10.3389/fcdhc.2022.1034698

**Published:** 2023-01-23

**Authors:** Xiaofan Jia, Liping Yu

**Affiliations:** Barbara Davis Center for Diabetes, University of Colorado School of Medicine, Aurora, CO, United States

**Keywords:** type 1 diabetes, autoantibodies, screening, prediction, biomarkers

## Abstract

While worldwide prevention efforts for type 1 diabetes (T1D) are underway to abrogate or slow progression to diabetes, mass screening of islet autoantibodies (IAbs) in the general population is urgently needed. IAbs, the most reliable biomarkers, play an essential role in prediction and clinical diagnosis of T1D. Through laboratory proficiency programs and harmonization efforts, a radio-binding assay (RBA) has been well established as the current ‘gold’ standard assay for all four IAbs. However, in view of the need for large-scale screening in the non-diabetic population, RBA consistently faces two fundamental challenges, cost-efficiency and disease specificity. While all four IAbs are important for disease prediction, the RBA platform, with a separate IAb test format is laborious, inefficient and expensive. Furthermore, the majority of IAb positivity in screening, especially from individuals with single IAb were found to be low risk with low affinity. It is well documented from multiple clinical studies that IAbs with low affinity are low risk with less or no disease relevance. At present, two non-radioactive multiplex assays, a 3-assay ELISA combining three IAbs and a multiplex ECL assay combining all four IAbs, have been successfully used as the primary methods for general population screenings in Germany and the US, respectively. Recently, the TrialNet Pathway to Prevention study has been organizing an IAb workshop which aims to analyze the 5-year T1D predictive values of IAbs. A T1D-specific assay with high efficiency, low cost and requiring low volume of sample will definitely be necessary to benefit general population screening.

Type 1 diabetes (T1D) is one of most common chronic diseases, often initiated in childhood. As of 2017, estimated global numbers of incidence and prevalence of T1D cases reached 234,710 and 9,004,610, respectively (1). The prevalence of T1D is increasing worldwide, 3-5% annually, with rates doubling every 20 years, especially in young children (2, 3) and even in youth (4). T1D can occur at any age but tends to develop in childhood (5) as the name ‘juvenile diabetes’ implies. In the US, 1.6 million people have T1D and as many have islet autoantibodies (IAbs) with high risk or preclinical T1D with normal blood glucose levels. In view of the natural history of T1D, once symptoms appear, beta cell mass has already reached a critical threshold (usually a residual 20–30% of normal amounts) (6), representing a very late phase of the disease. Even with the most advanced medical care in America, the prevalence of diabetic ketoacidosis (DKA) at T1D diagnosis is still as high as 40% with a 2% annual increase (7). Severe hypoglycemia and DKA are associated with high mortality rates, particularly in younger patients (8). And over a whole lifetime, diabetic complications continue to be a major cause of morbidity and mortality in persons with T1D (9). However, T1D is predictable. There is a long prodromal phase with months to years before symptoms develop, which leaves opportunities for disease prediction, and possibly prevention. Individuals at risk for T1D need to be identified before the onset of symptoms, to prevent life-threatening DKA and related morbidities and mortality (10, 11). Individuals need to be identified for preventive trials to reverse or slow down the progression to overt clinical T1D (12), and emotional support and education needs to be provided to reduce psychologic stress at diagnosis (10). As well, the underlying mechanism of islet autoimmunity and potential triggers need to be defined (13).

Although T1D mainly results from T-lymphocyte mediated destruction of insulin producing beta cells within pancreatic islets, appearance of IAbs that develop years before clinical disease are currently the most reliable biomarkers for T1D prediction and clinical diagnosis. Early T1D screening studies like Diabetes Prevention Trial-type 1 (DPT-1, continuing as current T1D TrialNet), Type 1 Diabetes Prediction and Prevention (DIPP), BABYDIAB, Diabetes Autoimmunity Study in the Young (DAISY) and the Environmental Determinants of Diabetes in the Young (TEDDY), aimed to understand the natural history of T1D by focusing on relatives of patients with T1D or genetically high-risk children in the general population. Familial clustering is a common feature of T1D with the risk of disease being 15-fold higher in families with T1D. However, the vast majority of children are diagnosed with the sporadic form of diabetes and the proportion of children with an affected first-degree relative at the time of diagnosis of T1D is only ∼10-20% (14). Worldwide prevention efforts for T1D are underway and multiple candidate interventions are being proposed to abrogate or slow progression to diabetes among high-risk individuals positive for IAbs. Mass screenings of children in the general population have been initiated in multiple countries (10, 11) and many plans are underway. Very recently, a two-age screening approach was proposed for early prediction of T1D (15) at ages 2 and 6 years in children, which is estimated to be capable of identifying the majority of children who will develop T1D by age 15 years and is likely to succeed in public health settings.

Since islet cell antibodies (ICA) were discovered in patients with autoimmune polyendocrine syndrome by an indirect immunofluorescence technique in 1974 (16, 17), four biochemically defined autoantibodies have been identified and well characterized including autoantibodies to insulin (IAA) (18), glutamic acid decarboxylase-65 (GADA) (19), insulinoma-associated antigen-2 (IA-2A) (20, 21) and zinc transporter-8 (ZnT8A) (22). A 3-stage classification of T1D based on these IAbs as the reliable biomarkers, has recently been proposed (23) and was widely accepted. Children with two or more of these IAbs are classified as T1D stage 1. Further progress to impaired metabolism with glucose intolerance or dysglycemia is classified as stage 2, and stage 3 is featured by the onset of typical clinical T1D. Almost all children who develop two or more IAbs will eventually progress to clinical T1D without consideration of HLA genotypes and the data is remarkably consistent across populations (24). At present, the ‘gold’ standard method to detect these four major IAbs is a radio-binding assay (RBA) which has been accepted worldwide and used for most past and current national and international T1D clinical trials. In the last two decades, the assay sensitivity and specificity of the RBA method have been greatly improved through laboratory proficiency programs (25, 26) and harmonization efforts (27). However, during past multiple T1D screening studies and currently with the need of large-scale screening in the general population, the RBA method faces two fundamental challenges: cost-efficiency and disease specificity.

All four biochemically defined IAbs, IAA, GADA, IA-2A and ZnT8A, have been shown to be important in the prediction and evaluation of the risk of progression to T1D in both relatives of patients with T1D and the general population. Mass screening of the general population for four IAbs with RBA where each IAb is measured individually is laborious and inefficient with a high cost and a large volume of blood required. A high throughput multiplex assay platform to combine the four major IAb assays in one with a lower cost is urgently needed to meet the current need of large-scale of screening in the general population. At present, two non-radioactive multiplex assays have been successfully used as the primary methods for general population screenings, a modified ELISA-based Elisa RSR™ 3 Screen ICA™ in the Fr1da study in Germany (28)and a multiplex electrochemiluminescence (ECL) assay (29) in the ASK study in the US. Both of these multiplex platforms have shown good sensitivity and specificity compared with the standard RBA and have successfully achieved their study goals of screening for pre-T1D subjects in the general population with high efficiency and low cost. The 3 Screen ICA ELISA used in Fr1da study (28) is a combination assay for measuring three IAbs (GADA, IA-2A, and ZnT8A) in one single well, but is not able to distinguish which of the three IAbs are present, and as a result, each positive signal needs to be retested by its corresponding single RBA for confirmation. However, the biggest disadvantage of the 3 Screen ICA ELISA is its inability to include autoantibodies directed against insulin (IAA). IAA have a very high rate of positivity in young children and are considered one of the first IAb to develop in children with T1D. The ELISA method does not achieve good sensitivity and specificity for IAA testing so far. The multiplex ECL assay used in the ASK study (29) combines 6 antibody tests in a single well, including all four IAbs, transglutaminase autoantibodies (TGA) for celiac disease, and COVID-19 antibodies. The multiplex ECL assay platform also has the advantage of easily building customized multiplexing panels to combine different antibody assays (up to 10) in one according to the need of a clinical setting. The limitation of the ECL assay platform is the requirement for a special plate reader. A new multiplex agglutination-PCR (ADAP) autoantibody assay method was reported very recently (30) and it combines four IAbs and TGA in one single well. The ADAP assay achieved good sensitivity and specificity in a study with a group of T1D patients vs healthy controls, however improvement on IA-2A and ZnT8A is still needed according to the data reported. Importantly, further validation of the ADAP assay for its performance, especially in non-diabetic population screening, will be expected. As well documented, up to 40% of T1D patients develop an additional autoimmune disorder (31, 32) and unfortunately, there is no easy and inexpensive tool to screen for these conditions. Integration of additional autoantibodies in multiplexed assay panels could be important for a successful screening strategy to benefit the clinical care of T1D patients. In the two T1D clinical trials DAISY and TEDDY, all study participants are screened for TGA for celiac disease. Persistent TGA positivity and celiac disease are secondary endpoints in both studies (33, 34). The International Society for Pediatric and Adolescent Diabetes (ISPAD) recommends screening for thyroid autoantibodies every 2 years after the diagnosis of T1D (35). Prevalence of both celiac and autoimmune thyroid disease are found to be higher than that of IAbs and participating parents and pediatric providers ranked the combined screening for T1D and other common autoimmune diseases as more valuable and attractive than screening for T1D alone (11).

In screening for relatives of T1D patients or for the general population, single IAb positivity is dominant. Children with a single T1D IAb had an extremely low risk. Only 14.5% progressed to clinical T1D during the 15 year follow-up (24). People in the field have been puzzled over the past decades about why these single IAbs have so poor a predictive value, while detection method of RBA shows highly specific, 98-99% in multiple Islet Autoantibody Standardization Program (IASP) workshops for many years. This not only caused a lot of confusion but paid high-cost efforts to longitudinally follow a large number of these children with single IAb in multiple clinical trials. Children with single IAb positivity are supposed to be in the early stage of islet autoimmunity, which would provide a better opportunity for early intervention to reverse or stop the autoimmune process. Unfortunately, single IAb positivity is unable to be integrated into T1D staging classification due to such a poor predictor of progression to disease and children with single IAb are excluded from almost all T1D clinical prevention studies. A large proportion of IAbs detected by RBA in initial screening, mainly in those individuals positive for a single IAb, were found to be low affinity. It is well documented in multiple studies that low-affinity IAbs are low risk and often not associated with disease (36–41). Most of them disappear during the longitudinal follow-up behaving as ‘transient’ positivity. These low-affinity IAbs detected by RBA, no doubt, are truly positive biochemically and the positive signals can be completely absorbed by native antigen molecules. Obviously, a high assay specificity is not necessary to represent a high disease specificity. Assay specificity of antibody measurement is commonly defined as the ability of an assay to score a positive result when the serum sample contains an antibody that can bind and/or neutralize the target molecule. Disease specificity, in terms of pre-clinical disease screening, refers to truly disease predictive values of antibodies detected in the assay. In a traditional concept, assay specificity is often referenced as a direct measure for disease specificity. Conventional antibody workshops determine whether antibodies are truly present or absent in the samples while often ignoring the qualities or categories of antibodies like binding affinity, IgG subclasses, etc. that have close association with the disease. To improve the performance of immunoassays measuring IAbs and to harmonize the results between laboratories, the IASP (previously DASP) has put in great effort and has been making great progress in organizing international IAb workshops for interlaboratory comparison studies every 18 months. IASP is so far the only official, internationally accepted workshop for IAbs supported by the Immunology and Diabetes Society (IDS) and NIH/NIDDK. All samples used in IASP workshop are limited to T1D cases and normal controls from which diagnostic values of IAbs for clinical patients vs healthy people can be estimated, while the predictive values of IAbs detected in non-diabetic population are not able to be evaluated. To overcome this shortfall, an IAb workshop sponsored by NIH/NIDDK, through the TrialNet-T1D Pathway to Prevention, has been organized with a large number of previously collected samples from relatives of T1D patients. The goal of this workshop is to test the 5-year predictive values of IAbs for clinical T1D and to identify newly developed testing methods, compared with the standard RBA. For the last decade, people have put great effort into seeking new assay methods to improve disease prediction. This has mainly been done by modifying the antigen constructs to remove the potentially low-affinity antibody binding sites or by adopting new technologies and assay platforms to discriminate high-affinity from low-affinity antibodies. GADA to N-terminally truncated (amino acids 96-585 (42) or 143-585 (43)) GAD65 (t-GADA) were found to be of higher disease prediction than GADA to full-length GAD65 in preclinical cohorts of first-degree relatives without the loss of sensitivity in T1D patients (44). In individuals with adult-onset diabetes, presence of t-GADA is associated with the clinical phenotype of T1D and predicts insulin therapy (45). Consistently, the ECL assay for IAbs has demonstrated its unique high-affinity antibody binding feature with effectively removing those low-affinity IAbs, leading to significantly higher predictive values for IAbs detected in multiple clinical trials like TrialNet-T1D Pathway to Prevention, TEDDY, Daisy and ASK (38–41). In DAISY, over 50% of the children, whose single IAb in RBA was confirmed by high-affinity ECL assay (n = 83), progressed to T1D in 10 years. In contrast, none of the 65 children, who were single IAb positive by RBA but negative by ECL assay, progressed to diabetes (46). In an ancillary TrialNet study (47), subjects who were positive for a single IAb by RBA but negative by ECL assay showed no worsening of glycemia, similar to subjects negative for all IAbs, during a median follow-up of 4.7 years. In contrast, glycemia worsened significantly in subjects with a single IAb confirmed by ECL assay, comparable with the worsening in subjects with multiple IAbs; the latter group had a higher progression to T1D (30%). In an ongoing Autoimmunity Screening for Kids (ASK) study screening general population children in Colorado, USA, as high as 80% of single IAb positivity generated by RBA were found to be ECL negative with low-affinity IAbs (48). Remarkably, high affinity IAbs confirmed at a patient’s very first initial positive visit stayed high affinity consistently over time (49). Similarly, those who were negative by ECL assay and showed low affinity at initial screening stayed low over time. No converting events from low to high or high to low affinity were seen over time. These results imply that high disease specific IAbs can be pre-identified in the early stage of initial screening using a high affinity assay. In addition, multiple studies have shown that at least 10% of Type 2 diabetes (T2D) patients are IAbs positive and a correct diagnosis in clinic is challenging, as the clinical phenotype does not initially require insulin for treatment. Anecdotal horror stories are prevalent of adult T1D patients misdiagnosed as having T2D and treated with oral hypoglycemic agents until severe DKA ensued. More often, insulin treatment is unnecessarily delayed, leading to further loss of endogenous insulin secretion and worse clinical outcomes compared to those patients receiving appropriate insulin therapy at diagnosis. The barriers to correctly diagnosing the type of diabetes present include limited access to high-quality IAb assays and a poor understanding of the natural history of autoimmune diabetes among healthcare providers. There are approximately 30M individuals with T2D in the United States (50) and the number increases annually. For identifying T1D in adult-onset diabetes, both the standard RBA and the ELISA based assay were reported to be problematic for disease specificity (48, 51), resulting in difficultly differentiating between true positives (T1D) and false positive (T2D). To correctly diagnose the type of diabetes, more disease specific IAbs need to be identified.

In summary, while worldwide prevention efforts for T1D are underway and multiple candidate interventions are being proposed to abrogate or slow progression to diabetes among high-risk individuals, mass screening of IAbs in the general population using the current standard assay method of RBA faces two fundamental problems of cost-efficiency and disease specificity. RBA with its single-antibody test platform for four major IAbs is laborious, inefficient, expensive, and the method is incapable of differentiating high-affinity (high-risk) from a large proportion of low-affinity IAbs (low-risk) results with poor disease specificity. The ELISA based assay may encounter the same challenges. To meet the needs of large-scale screening in the general population, new IAb assay platforms should have the potential to incorporate high affinity measurements with high efficiency (multiplexing). Recently, TrialNet-T1D Pathway to Prevention study has organized an IAb workshop which aims to analyze 5-year T1D predictive values of IAbs detected in non-diabetic subjects, either single or multiple IAbs. These efforts will enrich our ability to identify high risk individuals more accurately and efficiently at an early stage and promote the advances of early intervention to the benefit of public health.

## Author contributions

All authors listed have made a substantial, direct, and intellectual contribution to the work and approved it for publication.
